# Pervasive admixture between eucalypt species has consequences for conservation and assisted migration

**DOI:** 10.1111/eva.12761

**Published:** 2019-02-12

**Authors:** Brenton von Takach Dukai, Cameron Jack, Justin Borevitz, David B. Lindenmayer, Sam C. Banks

**Affiliations:** ^1^ Fenner School of Environment and Society The Australian National University Canberra Australian Capital Territory Australia; ^2^ ANU Bioinformatics Consultancy, John Curtin School of Medical Research Australian National University Canberra Australian Capital Territory Australia; ^3^ Research School of Biology The Australian National University Canberra Australian Capital Territory Australia; ^4^ Centre of Excellence in Plant Energy Biology The Australian National University Canberra Australian Capital Territory Australia; ^5^ Research Institute for the Environment and Livelihoods Charles Darwin University Darwin Northwest Territories Australia

**Keywords:** admixture, eucalypt, *Eucalyptus regnans*, gene flow, hybrid, mountain ash, population genetics, SNP

## Abstract

Conservation management often uses information on genetic population structure to assess the importance of local provenancing for ecological restoration and reintroduction programs. For species that do not exhibit complete reproductive isolation, the estimation of population genetic parameters may be influenced by the extent of admixture. Therefore, to avoid perverse outcomes for conservation, genetically informed management strategies must determine whether hybridization between species is relevant, and the extent to which observed population genetic patterns are shaped by interspecific versus intraspecific gene flow. We used genotyping by sequencing to identify over 2,400 informative single nucleotide polymorphisms across 18 populations of *Eucalyptus regnans* F. Muell., a foundation tree species of montane forests in south‐eastern Australia. We used these data to determine the extent of hybridization with another species, *Eucalyptus obliqua* L'Hér., and investigate how admixture influences genetic diversity parameters, by estimating metrics of genetic diversity and examining population genetic structure in datasets with and without admixed individuals. We found hybrid individuals at all sites and two highly introgressed populations. Hybrid individuals were not distributed evenly across environmental gradients, with logistic regression identifying hybrids as being associated with temperature. Removal of hybrids resulted in increases in genetic differentiation (*F*_ST_), expected heterozygosity, observed heterozygosity and the inbreeding coefficient, and different patterns of isolation by distance. After removal of hybrids and introgressed populations, mountain ash showed very little population genetic structure, with a small effect of isolation by distance, and very low global *F*_ST_(0.03). Our study shows that, in plants, decisions around provenancing of individuals for restoration depend on knowledge of whether hybridization is influencing population genetic structure. For species in which most genetic variation is held within populations, there may be little benefit in planning conservation strategies around environmental adaptation of seed sources. The possibility for adaptive introgression may also be relevant when species regularly hybridize.

## 1 INTRODUCTION

Substantial biodiversity declines are occurring in many regions of the world due to widespread land clearing, habitat degradation, introduced species and climate change (Evans et al., 2011; Pounds et al., 2006; Woinarski, Burbidge, & Harrison, 2015). Extensive and ongoing land clearing has led to major reductions in forest cover globally (Achard et al., 2014; Reside et al., 2017; Taubert et al., 2018), with synergistic interactions between stressors placing some ecosystems under high threat of rapid collapse or changes in ecosystem state (Brook, Sodhi, & Bradshaw, 2008; Lindenmayer, Hobbs, Likens, Krebs, & Banks, 2011; Lindenmayer & Sato, 2018). With such widespread changes facing ecosystems, it is critical to understand how these stressors interact with the fundamental ecological processes operating within and between foundation species, to adequately manage biodiversity across landscapes.

Using genetic approaches to inform management activities allows conservation efforts to be targeted towards sites of unique genetic composition or adaptive importance, making population genetic studies valuable in many taxa (Ikeda et al., 2017; Maunder, Cowan, Stranc, & Fay, 2001; McCartney‐Melstad & Shaffer, 2015; Reynolds et al., 2015). To maximize beneficial outcomes, it is vital that our understanding of the population genetic diversity and structure in target species is as accurate as possible, particularly in the context of a changing environment. For example, understanding patterns of local adaptation across the range of a species is important for developing methods of assisted gene flow to mitigate the impacts of climate change and other threatening processes (Kelly & Phillips, 2016; Supple et al., 2018).

Genetically informed conservation requires a detailed understanding of the spatial distribution of genetic diversity, particularly as it relates to environmental adaptation. Spatial genetic structure and population genetic differentiation are typically considered to be driven by the influences of gene flow, genetic drift and local adaptation (Orsini, Vanoverbeke, Swillen, Mergeay, & Meester, 2013). However, for species that do not exist in complete reproductive isolation, the estimation of population genetic parameters may be influenced by the extent of hybridization and introgression with closely related species. This could have large implications for the application of genetic data to conservation management of species, for example, by committing resources to putatively distinct populations, when they may actually contain highly admixed individuals.

With the advent of modern DNA genotyping techniques, studies investigating thousands of genetic markers from across the genome are becoming more common (Gaughran et al., 2018; Hand et al., 2015; Harvey, Aleixo, Ribas, & Brumfield, 2017; Hudson, Freeman, Myburg, Potts, & Vaillancourt, 2015), and several studies have investigated patterns of nuclear genetic structure and gene flow across large geographic regions (Hecht, Matala, Hess, & Narum, 2015; Hendricks et al., 2017; Sampson et al., 2018; Shriver et al., 2005). Studies such as these provide critical information for the conservation of populations with unique genetic heritage, identification of areas of adaptive potential for assisted migration and location of source populations or historical refugia (Hecht et al., 2015; Supple et al., 2018).

Australian natural vegetation communities are dominated by the hyperdiverse and commercially important tree genus *Eucalyptus* L'Hér. With about 700 species recognized (Bayly, 2016), eucalypts are an integral part of the Australian landscape and are foundation species in many ecological communities. For such an important component of Australia's vegetation, there is still much to understand about gene flow, population dynamics and genetic structure in eucalypts. Gene flow in plants is typically the result of both pollen and seed dispersal, with pollen typically playing a greater role in eucalypts because it tends to disperse further than seeds (Barber, 1965; Petit et al., 2005; Potts & Wiltshire, 1997). Comparisons of the maternally inherited chloroplast and biparentally inherited nuclear DNA have shown that pollen‐mediated gene flow can be up to at least 200 times greater than seed‐mediated gene flow in some species (Bloomfield, Nevill, Potts, Vaillancourt, & Steane, 2011; Nevill, Bradbury, Williams, Tomlinson, & Krauss, 2014), although at least one study found that gene flow from seed dispersal is practically equivalent to that from pollen dispersal (Jones, Shepherd, Henry, & Delves, 2006).

Given the importance of understanding population genetic structure for conservation, and the knowledge that hybridization in eucalypts is a widespread and common phenomenon (Griffin, Burgess, & Wolf, 1988), we investigated these two aspects in *Eucalyptus regnans* F. Muell., (mountain ash) one of Australia's most well‐known and economically important trees. The existence of hybrids between *E. regnans* and the frequently co‐occurring *Eucalyptus obliqua* L'Hér. (messmate stringybark) has long been known (Ashton, 1956); however, the extent of hybridization across the range of the species has never been investigated. Similarly, while the chloroplast genetic structure of *E. regnans* has been studied (Nevill, Bossinger, & Ades, 2010), the structure of the nuclear genome has not. To address these knowledge gaps, our aims were to (a) identify the extent and possible drivers of hybridization across the geographic distribution of *E. regnans*, (b) describe how identification of admixture using genomic data may influence our understanding of population genetic structure and (c) consider how these factors would influence current management strategies in eucalypts. We address these aims using genotyping by sequencing to obtain large numbers of genomewide genetic markers on individual samples across the natural geographic range of the species. We predict that (a) some individuals and populations will show greater levels of admixture with *E. obliqua*, (b) levels of admixture will be driven in part by local environmental variables and (c) the inclusion or exclusion of hybrid individuals in population genetic analyses will lead to different strategic outcomes for management. If these predictions are true, there are implications for future studies of population genetic structure and the planning of restoration plantings and assisted gene flow.

## 2 MATERIALS AND METHODS

### 2.1 Study area and species

*Eucalyptus regnans* grows in wet forests of the south‐east Australian states of Victoria and Tasmania. It is the tallest angiosperm in the world, with reliable records of individuals exceeding 100 m (Beale, 2007; Hardy, 1918, 1935). It is also a serotinous obligate seeder, requiring high‐intensity fires to open the understorey, create fertile ash beds and stimulate the mass release of seeds from the forest canopy (Ashton1981b; Ashton & Chinner, 1999). Without fire, trees are typically unable to produce offspring that survive to maturity, primarily due to predation of seeds by ants (Ashton, 1979; O'Dowd & Gill, 1984), low availability of light (Gilbert, 1959), browsing of seedlings by herbivores and fungal infection of seedlings (Ashton & Macauley, 1972).

*Eucalyptus regnans* is patchily distributed through a 700 km by 500 km area, growing only where climatic conditions are suitable (Cochrane, 1969). It reaches its highest elevations (>1,100 m ASL) in the northernmost part of its range, on the Errinundra Plateau, and grows near to sea level in some southern parts of its Tasmanian distribution. As the island of Tasmania has been separated from the Australian mainland by more than 200 km for over ten thousand years (Duncan, Worth, Jordan, Jones, & Vaillancourt, 2016; Lambeck, Rouby, Purcell, Sun, & Sambridge, 2014), it is assumed that there has been very little or no gene flow between *E. regnans *stands in these two regions for at least that length of time.

In a number of locations throughout Victoria and Tasmania, trees displaying intermediate characteristics between *E. regnans* and other species have been recorded. These specimens have been identified mostly as hybrid individuals between *E. regnans* and *E. obliqua*, and, less commonly, *E. regnans* and *E. macrorhyncha *(Ashton, 1958, 1981a; Ashton & Sandiford, 1988). At least two individuals have also been found that appear to be tri‐hybrids—the result of a *E. regnansx obliqua *hybrid individual mating with a *E. macrorhyncha *(Yorke & Ashton, 1982). As red stringybark does not occur naturally in Tasmania, *E. regnans × macrorhyncha* hybrids do not occur there.

### 2.2 Sample collection

**Figure 1:**
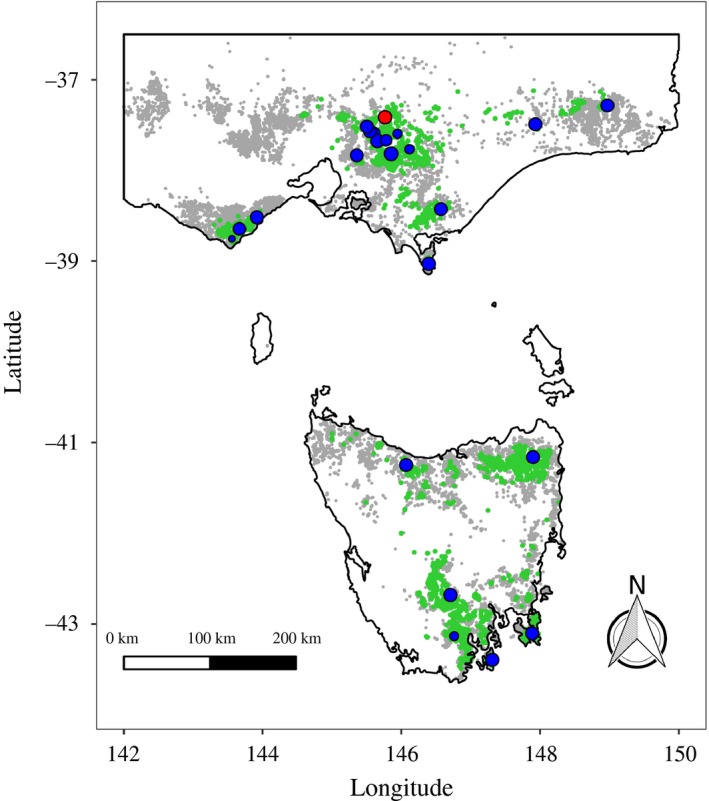
Map showing the overall distribution of *Eucalyptus regnans* (green shading) and *Eucalyptus obliqua* (grey shading) in the Australian states of Victoria and Tasmania, and the locations visited for collection of tissue samples for genotyping by sequencing. Species distributions are derived from records of each species found on the Atlas of Living Australia. The blue circles represent sites where *E. regnans* samples were collected, and the red circle represents the site where *E. obliqua* samples were collected. The number of samples collected at each site is indicated by the size of the circle

We collected 387 *E. regnans* tissue samples from across its geographic distribution (Figure 1). At each of 16 sites, we walked a transect collecting tissue from trees spaced at least 20 m apart, until we had sampled 20 trees. We targeted trees with diameters at breast height of more than 60 cm, to avoid sampling younger trees that were propagated after the practice of reseeding logged coupes using seed of nonlocal provenance became common practice (Flint & Fagg, 2007). As the combined effects of logging and wildfires have caused a reduction in the size and frequency of old undisturbed patches of trees (Lindenmayer, Blanchard, Blair, McBurney, & Banks, 2016), a linear transect of fixed length was sometimes impossible. At seven of the sites, we collected a second sample from the 20th tree, to serve as a technical replicate from the field. We were also able to incorporate an extra 42 *E. regnans* samples collected during fieldwork for other studies into some analyses, taken from various locations (Supporting Information Table S1). Twenty‐one *E. obliqua* samples, taken from the Cathedral Range region in Victoria, were also sequenced to allow us to determine the extent of hybridization between the two species. All samples were putatively identified as *E. regnans* or *E. obliqua* using purported diagnostic morphological characters (Brooker & Kleinig, 2006).

Tissue collected was mostly cambium, obtained by cutting through the rough and smooth bark using a machete, and slicing off a 10 × 5 × 0.2 cm strip. A small number of samples were leaf tissue, collected by climbing trees using standard arborist techniques and picking two fresh, growing leaves. All samples were air‐dried in individual brown paper bags and then stored at 4°C prior to DNA extraction.

### 2.3 Sample preparation and sequencing

Approximately 600 mg of each tissue sample was chilled to −65°C and homogenized using an automated tissue grinding machine (Labman Max Planck Cryogenic Grinder Dispenser, Labman Automation & Custom Robotics), before storage at −18°C to await DNA extraction.

Samples were ordered randomly, and whole genomic DNA was extracted in plate format by following the kit (Stratec Invisiorb DNA Plant HTS 96) instructions. Library preparation for genotyping by sequencing included (a) digestion using PstI restriction enzyme (New England BioLabs, Inc.), (b) ligation using T4 DNA ligase (New England BioLabs, Inc.), (c) a purification step (Qiagen MinElute 96‐well PCR purification kit), (d) PCR amplification using two GBS primers (Integrated DNA Technologies), (e) postpurification quantitation using microfluidic capillary electrophoresis (PerkinElmer LabChip GX II), (6) pooling of 12 ng DNA per sample using an automated robotic liquid handling machine (PerkinElmer NGS Express) and (f) a final purification step (Sigma‐Aldrich Genelute PCR Clean‐Up Kit).

Size fractionation, 250–450 base‐pair gel cutout, and sequencing was conducted at the Australian Cancer Research Foundation Biomolecular Resource Facility (BRF) at the John Curtin School of Medical Research (Australian National University) on portions of four lanes (grouped with other *E. regnans* sequencing experiments) of an Illumina HiSeq 2500 machine using a 100‐base paired‐end read.

### 2.4 Demultiplexing and initial filters

Of the 408 samples (387 *E. regnans* and 21 *E. obliqua*), sequencing resulted in nearly 1.49 billion read pairs. We demultiplexed reads using exact matches and combinatorial index mode with Axe (Murray & Borevitz, 2018) and were unable to assign 7% of read pairs to a sample. We then used BBDuk to remove adapters and quality‐trim (Phred score *Q* = 30) reads at both ends, and NextGenMap (Sedlazeck, Rescheneder, & von Haeseler, 2013) to align reads to the *E. grandis* v2.0 reference genome (Bartholome et al., 2015; Myburg et al., 2014). We used SAMtools (Li et al., 2009) to convert the dataset into sample‐specific Binary Alignment/Map (BAM) files and sort reads.

To create a sample‐by‐SNP matrix, we used the “ANGSD” software package to first calculate genotype likelihoods (McKenna et al., 2010) and used these likelihoods to call genotypes. Loci were initially filtered based on (a) a probability of at least 99.999% that the site was variable, (b) the site was genotyped in at least 50 individuals, (c) the site had a minimum average sequencing depth per sample of 0.5 and (d) the site had a maximum average sequencing depth per sample of 1,000. Genotype likelihoods were retained and exported in BEAGLE file format for admixture analysis using “NGSadmix” (Skotte, Korneliussen, & Albrechtsen, 2013). Called genotypes were used for the remainder of the analyses and were derived from the likelihoods based on a posterior genotype probability (≥0.95) and assuming a uniform prior. This produced a matrix containing 408 samples and 49,622 SNPs, where the mean and median read depth per site per sample were 28.3 and 9.9, respectively.

Two separate filtering strategies were conducted on this dataset using the statistical software package R (R Core Team, 2017). The first of these developed a set of SNPs for investigating the extent of admixture with *E. obliqua*. The second method was used to investigate whether hybridization influences population genetic structure and isolation by distance across the geographic distribution of *E. regnans*. Each of these filters has been discussed in the methods of the relevant analysis.

### 2.5 Extent of admixture

We investigated individual admixture proportions using three techniques and averaged the results to improve accuracy and reliability. Firstly, the Bayesian clustering method in STRUCTURE v2.3.4 (Falush, Stephens, & Pritchard, 2003; Pritchard, Stephens, & Donnelly, 2000) was used, with a 50,000 burn‐in and 200,000 Markov chain Monte Carlo (MCMC) iterations, a *K* value of 2, using an admixture model and correlated allele frequencies. To obtain SNPs used in this analysis, we filtered on call rate (≥66% of samples genotyped) and minor allele frequency (MAF ≥ 0.01), retaining 2,192 SNPs. Samples missing more than two‐thirds of these loci were removed from any further analysis, retaining 380 samples. Next, we used an eigen‐analysis approach to investigate individual ancestries, using the *snpgdsAdmixProp* function of the “SNPRelate” package (Zheng et al., 2012; Zheng & Weir, 2016), with the same 2,192 SNPs and 380 samples used for the STRUCTURE analysis. Lastly, we used the expectation–maximization algorithm in NGSadmix (Skotte et al., 2013), using the GATK genotype likelihoods (McKenna et al., 2010) produced by ANGSD, assuming two ancestral populations, and requiring the minor allele to be present in at least eight individuals, retaining 16,634 loci. Bar plots of admixture for all 380 samples allowed for visual comparison of each method. As the admixture proportions between the three methods were highly correlated (discussed in the Results section), we then averaged the admixture coefficients and used these mean values to exclude or retain individuals for the remaining analyses. Samples with a *E. obliqua* ancestry coefficient greater than 0.1 were considered hybrids, with coefficients of 0.4–0.6 indicating intermediate levels of hybridization, and 0.1–0.4 or 0.6–1 indicating closer affinity to *E. regnans* or *E. obliqua,* respectively (Field, Ayre, Whelan, & Young, 2009; Melville et al., 2017). While this method of identifying hybrid individuals is unlikely to have completely removed admixture from the study, we considered it sufficient to demonstrate how accounting for hybridization and introgression in the analysis pipeline can influence the results of, and conclusions drawn from, genetic analyses. It is unfeasible and possibly inappropriate to try and completely remove all admixture, with gene flow between the two species possibly occurring throughout their recent evolutionary history.

As principal components analysis (PCA) is often used as an initial method of removing outlier samples (Jordan, Hoffmann, Dillon, & Prober, 2017; Supple et al., 2018), we first checked whether PCA would be appropriate for the identification and removal of hybrid individuals from the dataset. We calculated pairwise Euclidean genetic distances using the 2,192 SNPs and 380 samples and then performed a PCA using the *indpca *function of the “hierfstat” (Goudet, 2005) package in R. The first two principal components for each sample were plotted using the “ggplot2” (Wickham, 2009) package, with samples coloured by the mean level of admixture with *E. obliqua*, as determined above.

Next, we calculated mean admixture proportions for every population and mapped this across the landscape using the “ggplot2” and “scatterpie” (Yu, 2018) packages.

### 2.6 Environmental association with admixture

The location of all 359 putative *E. regnans* samples was uploaded into the Atlas of Living Australia's (ALA) Spatial Portal (https://spatial.ala.org.au/#), and 15 environmental variables at each point were extracted. Environmental variables used in the ALA were collated or derived from various sources (De Vries, 2009; Williams et al., 2006, 2010; Xu & Hutchinson, 2011, 2013). Variance inflation factors were used to remove variables that showed multicollinearity, and visual inspection of histograms for each variable allowed removal of two variables that showed very little variation across all individuals. This left eight variables remaining, including mean annual rainfall (RAIN), mean annual solar radiation (RAD), historical (pre‐European) phosphorus availability (PHOS), topographic wetness index (TWI), mean maximum temperature of the hottest month (MAXTEMP), mean minimum temperature of the coldest month (MINTEMP), as well as topographic aspect, which was transformed into a north–south component (NORTH) and an east–west component (EAST). A binary response variable was also created that identified each sample as either a hybrid individual or a “pure” *E. regnans*. All predictor variables were scaled (by subtracting the mean and dividing by the standard deviation) prior to fitting any models.

To identify whether any predictor variables showed nonlinearity on the logistic scale, we fitted a binomial generalized additive model in R and plotted the component smooth functions for each variable on the scale of the linear predictor. The MAXTEMP variable showed signs of nonlinearity and so was transformed with the inverse reciprocal, which improved linearity substantially.

A binomial generalized linear mixed‐effects model was then run in R, using the *glmer* function of the “lme4” (Bates, Maechler, Bolker, & Walker, 2015) package, using the binary response variable, the seven unmodified environmental predictor variables and the new MAXTEMP predictor variable. The estimated tree age (AGE) for each sample was also included as a predictor to determine whether this was relevant, and a random effect of stand was fitted to account for repeated sampling. Overdispersion was checked using the model residuals and degrees of freedom, and spatial autocorrelation was investigated in the model residuals using visual examination of a variogram created with the *variog* function of the “geor” (Ribeiro Jr. & Diggle, 2016) package.

All possible submodels of the global model were fitted and ranked by Akaike's information criterion corrected for finite sample sizes (AICc) using the “MuMIn” (Barton, 2016) package. Models with AIC values ≤2 above the top‐ranked model were considered useful for inference (Hegyi & Garamszegi, 2011).

### 2.7 Influence of admixture on population structure

To determine the level of influence that unrealized hybridization can have on population structure, we first filtered SNPs using call rate (≥0.4), minor allele frequency (MAF ≥ 0.01) and observed heterozygosity (≤0.5). The likelihood that each SNP does not deviate from Hardy–Weinberg equilibrium (HWE) was checked using the *HWChisqStats* function of the “Hardy–Weinberg” (Graffelman, 2015) package. Any SNP out of HWE in more than three populations (where *n* ≥ 15) was removed from further analysis. In addition, the *snpgdsLDpruning* function in the “SNPRelate” (Zheng et al., 2012) package was used to prune out SNPs using a linkage disequilibrium threshold of 0.5 and a sliding window of 5,000 bp.

Filtering was done on two groups: (a) all samples including hybrids (but excluding the reference *E. obliqua* samples) and (b) pure *E. regnans* individuals (i.e., those with <10% admixture with *E. obliqua*). In the admixture‐inclusive group, 2,474 SNPs were retained. For the pure group, 2,481 SNPs were retained. Any samples with more than 50% missing data were removed from each dataset, leaving 323 samples and 228 samples in the admixture‐inclusive and admixture‐free groups, respectively. Visual inspection of hierarchical clustering dendrograms, created using the *hclust* function on a Euclidean genetic distance matrix, showed that all biological replicates were closely paired in both datasets (Supporting Information Figure S2), indicating they were reliable and contamination was not likely to be a factor.

Standard population genetic parameters were calculated for all loci in both groups, using the *basic.stats* function in the “hierfstat” (Goudet, 2005) package. To compare the influence of including hybrid samples on these parameters, the means and standard errors of the inbreeding coefficient (*F*_IS_), genetic differentiation (*F*_ST_), expected heterozygosity (*H*_E_) and observed heterozygosity (*H*_O_) were calculated across all loci and plotted as bar charts.

Prior to further analysis, all sites containing <10 individuals were removed from both datasets, leaving the pure dataset with 15 sites and the admixture‐inclusive dataset with 18 sites. Population genetic structure was assessed in both datasets using principal coordinates analysis (PCoA) of pairwise population genetic distances as well as through isolation by distance. Pairwise population Euclidean genetic distances were calculated by first converting genotypes to genpop objects using the *df2genind *and *genind2genpop* functions in the “adegenet” (Jombart, 2008) package, and then using the *dist.genpop* function to produce the distance matrix. Principal components were created using the *cmdscale* function, with populations plotted using the “ggplot2” package. To investigate isolation by distance, we calculated pairwise *F*_ST_ between populations using the “Nei87” method of the *genet.dist* function in the “hierfstat” package, and regressed these distances against pairwise population geographic distance, calculated using the *earth.dist* function in the “fossil” (Vavrek, 2011) package. Genetic differentiation and geographic distance were transformed to *F*_ST_/(1 − *F*_ST_) and log(geographic distance), respectively, to allow for linear interpretation (Rousset, 1997).

### 2.8 Spatial structure and population genetics in *Eucalyptus regnans*

Spatial structuring of genotypes was investigated using two methods; firstly, a Mantel test (Mantel, 1967) comparing pairwise genetic distance with the natural logarithm of pairwise geographic distance was performed using the *mantel.rtest* function in the R package “ade4” (Dray & Dufour, 2007). We then used the spatial structure analysis function in GenAlEx v6.503 (Peakall & Smouse, 2006, 2012) to determine the maximum geographic distance at which genotypes show significant spatial autocorrelation. This was interpreted as the maximum distance at which the lower confidence interval of the spatial autocorrelation coefficient *r *was greater than zero on the y‐axis.

To investigate population genetics within *E. regnans*, we calculated means and standard errors of the number of alleles (*A*), number of effective alleles (*A*_E_), *H*_O_, *H*_E_ and *F*_IS_ for each of the 15 populations. We then calculated the pairwise genetic distance between all individuals in these populations using the “Dch” method of the *genet.dist* function. This distance matrix was then read into GenAlEx to conduct an analysis of molecular variance (AMOVA) with 999 permutations to determine the amount of genetic variation explained within and among sites.

## 3 RESULTS

### 3.1 Extent of admixture

Despite NGSadmix using a different approach (likelihoods instead of called genotypes) and much larger number of loci, there was a very strong correlation of ancestry coefficients computed using that method and both STRUCTURE (*r* = 0.94, *p* < 0.001) and SNPRelate (0.95, *p* < 0.001), with bar plots showing near‐identical patterns between methods (Supporting Information Figure S1). There was an even stronger correlation between the results of STRUCTURE and SNPRelate (0.98, *p* < 0.001). Excluding the reference *E. obliqua* samples, SNPRelate, STRUCTURE and NGSadmix identified 59, 130 and 190 hybrid individuals (>10% admixture with *E. obliqua*), respectively. All hybrid samples identified in SNPRelate were also identified using STRUCTURE, but a small proportion of samples identified as hybrids in SNPRelate and STRUCTURE were not identified as such by NGSadmix. After calculating the mean value of the *E. obliqua* admixture coefficient for the three methods, 170 samples were identified as hybrids. Of these, 75 samples had *E. obliqua* admixture coefficients between 0.1 and 0.4, five had coefficients between 0.4 and 0.6, 11 had coefficients between 0.6 and 0.9, and 16 had coefficients of greater than 0.9.

**Figure 2:**
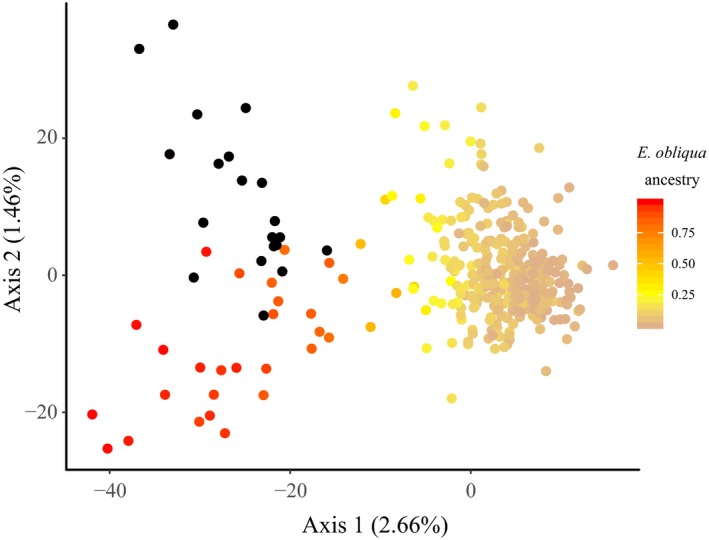
Principal components analysis of pairwise genetic distance between 380 putative *Eucalyptus regnans* and 20 putative *Eucalyptus obliqua* trees. *Eucalyptus regnans* was sampled from across the natural geographic range of the species. Euclidean genetic distances were calculated using 2,192 single nucleotide polymorphisms. Putative *E. regnans* samples are coloured by their proportion of admixture with *E. obliqua*. The reference *E. obliqua* samples are coloured black

Principal components analysis showed that highly admixed samples (e.g., those from Wilsons Promontory) were able to be easily identified due to their separation from the bulk of the *E. regnans* species cluster (Figure 2). Unfortunately, PCA methods were unable to clearly separate out individuals with low and moderate levels of admixture, which account for about two‐thirds of the admixed samples. Pairwise genetic distances involving these individuals presumably fall within the natural variation of genetic distance within *E. regnans*, meaning that distance‐based methods are more conservative in the identification and removal of hybrids.

**Figure 3:**
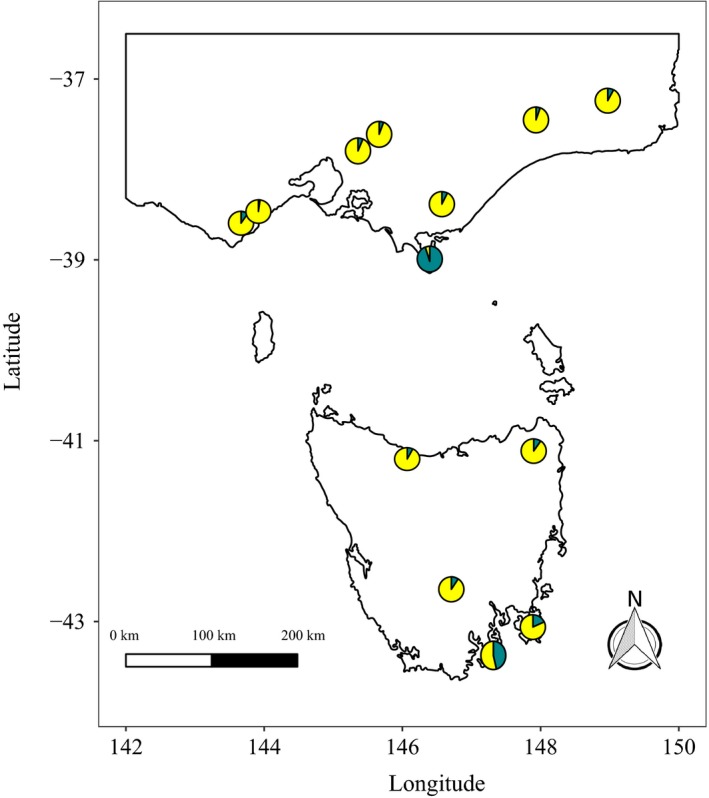
Map showing the mean proportion of admixture for 18 sites (with *n* > 10) where *Eucalyptus regnans* was sampled. Pie charts show the mean proportion of admixture between *E. regnans* (yellow) and *Eucalyptus obliqua* (turquoise). Six geographically close sites sampled in the central region of Victoria were not significantly different in the amount of admixture (one‐way ANOVA, *F*_5,108_ = 1.18, *p* = 0.324) and so have been pooled here for clarity

The mean proportion of hybrids per sample (at sites where more than 10 samples were analysed) was 0.24. Not a single site was completely free of hybrids, but seven sites had just one hybrid. The mean *E. obliqua *ancestry coefficient of a site was 0.11 (±0.21), with sites varying considerably in the amount of admixture (Figure 3). Samples taken from Wilsons Promontory showed the highest degree of admixture, with a mean *E. obliqua *ancestry coefficient of 0.87. The Tasmanian sites on Bruny Island and the Tasman Peninsula also had mean *E. obliqua *ancestry coefficients greater than 0.1, at 0.42 and 0.15, respectively.

### 3.2 Environmental association with admixture

**Figure 4:**
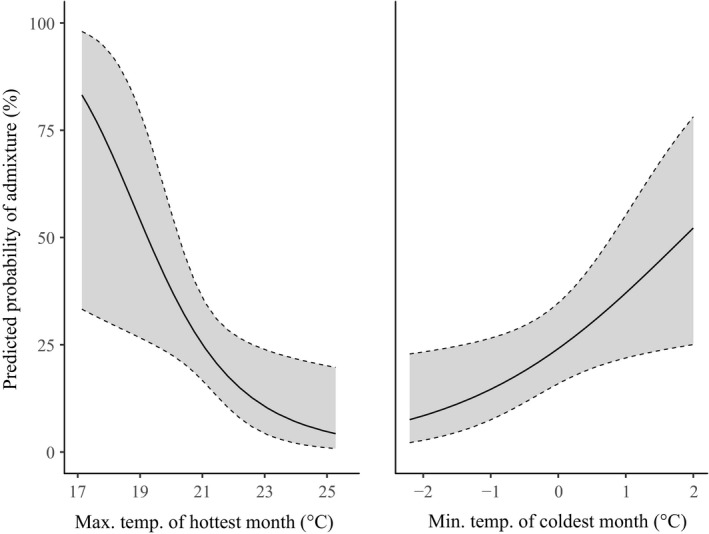
Effect plot showing the relationship of two predictor variables, the mean maximum temperature of the hottest month and the mean minimum temperature of the coldest month, with the probability that a *Eucalyptus regnans* individual will have >10% genetic admixture with *Eucalyptus obliqua*. Grey areas indicate 95% confidence intervals

Model selection showed that hybrid individuals were not randomly distributed across all environmental variables. Of the 11 top models (ΔAICc < 2), MINTEMP and MAXTEMP were identified in all 11, suggesting that these two variables had the strongest effects on the probability of hybrid occurrence. Sites with a high MAXTEMP (hot summers) and sites with a low MINTEMP (cold winters) had lower probabilities of hybrid occurrence, whereas sites with a high MINTEMP and sites with a low MAXTEMP had higher probabilities of hybrid occurrence (Figure 4). Variables that occurred in fewer top models included NORTH, RAD, PHOS, RAIN, AGE and TWI; however, none of these variables showed any clear trends with hybrid occurrence (Supporting Information Figure S3). EAST was not identified in any top models.

### 3.3 Influence of admixture on population structure

**Figure 5:**
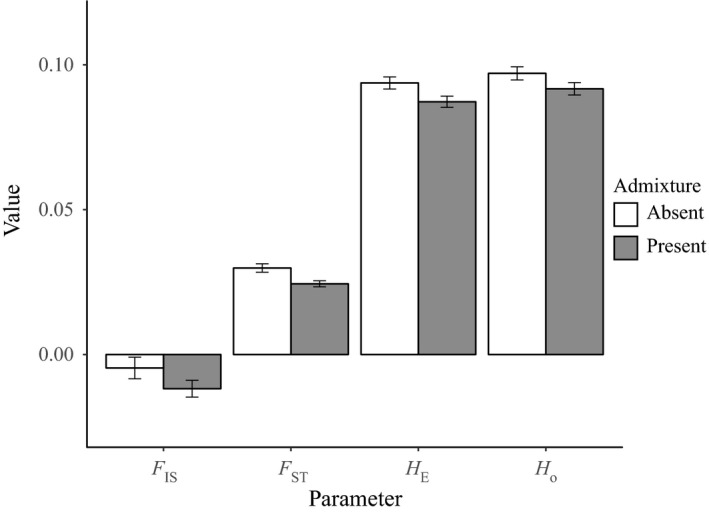
Comparison of genetic parameters, including the inbreeding coefficient (*F*_IS_), genetic differentiation (*F*_ST_), expected heterozygosity (*H*_E_) and observed heterozygosity (*H*_O_) between two datasets, one of which contains hybrid individuals (*n* = 323) and another where hybrid individuals have been removed (*n* = 228). The datasets were filtered using the same criteria, resulting in 2,474 single nucleotide polymorphisms when hybrids were included and 2,481 when hybrids were removed. Error bars represent standard errors of the mean

The removal of hybrid individuals from the dataset modified the values of the genetic parameters investigated (Figure 5), with the parameters *H*_E_, *H*_O_, *F*_ST_ and *F*_IS _increasing from 0.087, 0.092, 0.024 and −0.012 in the admixture‐inclusive dataset, to 0.094, 0.097, 0.03 and −0.005 in the admixture‐free dataset.

**Figure 6:**
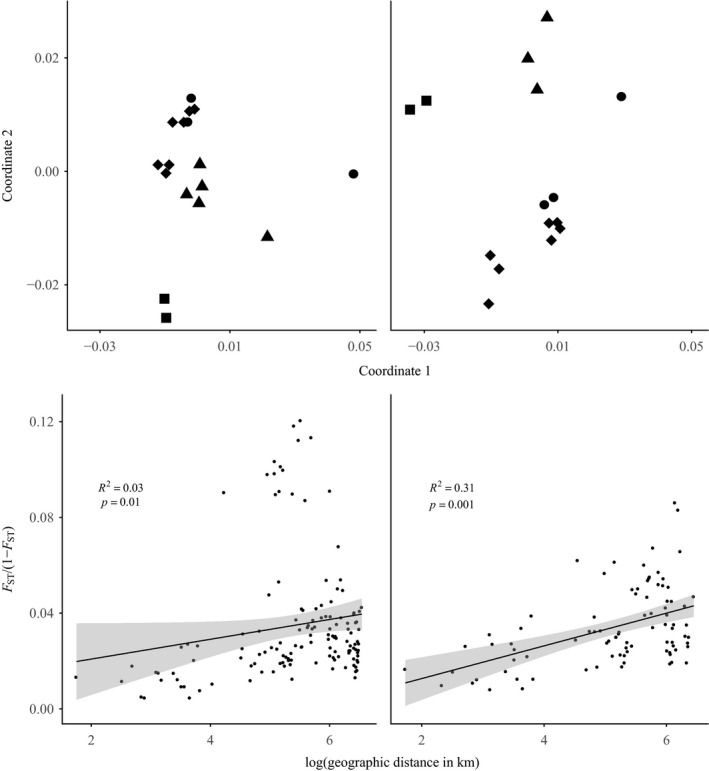
Plots of pairwise genetic distance and isolation by distance in *Eucalyptus regnans* populations across its natural geographic distribution. Plots on the left‐hand side of the figure include the presence of hybrid individuals and populations. Plots on the right‐hand side of the figure have had all hybrid individuals removed. The top two plots show the first two principal coordinates of Euclidean genetic distances, where squares, triangles, diamonds and circles, respectively, represent populations from the Otways, Tasmania, Central Victoria and Gippsland regions of Australia. The bottom two plots indicate the effect of increasing geographic distance on genetic differentiation, with both axes transformed to allow visualization using a linear relationship

Removal of hybrids led to greater resolution of geographic population structure in the PCoA of pairwise population genetic distances and a stronger pattern of isolation by distance from the Mantel test (Figure 6). In the admixture‐inclusive dataset, the first principal coordinate was associated with the degree of admixture, with Wilsons Promontory and Bruny Island separated out from the rest of the sites. The high level of admixture at Wilsons Promontory resulted in comparatively much higher pairwise *F*_ST_ values between this and other sites, leading to a reduction in fit of the isolation‐by‐distance model when this site was included in the analysis.

### 3.4 Spatial structure and population genetics in *Eucalyptus regnans*

After removing all hybrids and discounting sites that had <10 samples, 200 individuals from 15 sites were retained. The spatial genetic structure analysis in GenAlEx identified weak but significant autocorrelation (determined by inspection of bootstrap confidence intervals) between genotypes at pairwise distances of up to 90 km, with the autocorrelation coefficient *r* intercepting zero at 108 km. A Mantel test, comparing the logarithm of geographic distance with Nei's genetic distance for all pairs of samples, showed that there was a relatively small but significant (*r* = 0.18, *p* = 0.001) positive effect of isolation by distance.

Within‐population genetic structure at the 15 sites was very similar (Supporting Information Table S2), with little variation in *A*, *A*_E_, *H*_O_, *H*_E_, or *F*_IS_. Most sites had higher *H_O_* than *H_E_*, and all sites had negative *F*_IS _values, implying that outbreeding is the common condition throughout the species distribution. Global *F*_ST_ was very low (0.03), and approximately equal to the mean pairwise *F*_ST_ between sites (0.028), despite some large (>600 km) pairwise geographic distances (Figure 6). Pairwise *F*_ST_ values ranged from 0.003, between the geographically close Central Victorian sites of Powelltown and Toolangi South (39 km), to 0.063, between the geographically distant Errinundra Plateau and Gellibrand River (489 km).

Across the overall distribution of the species, *E. regnans* showed very little population structure, with the AMOVA indicating that just 3% of the genetic variation was occurring among populations, and 97% within populations. The first two coordinates of the PCoA explained 13.2% and 11.5% of the total variation, with the Tasmanian samples intermediate between sites in central‐eastern Victoria and sites from the Otways region (Figure 6). The PCoA also identified the Errinundra Plateau site as being relatively distinct from the rest of the sites.

## 4 DISCUSSION

Using a genomewide SNP dataset, we investigated the extent of hybridization between two widespread forest trees and the way in which admixture influences estimation of genetic parameters and interpretation of population genetic structure. We found that hybrids were not distributed evenly across geographic or environmental space, with some populations showing more admixture than others, and a strong association of hybrid occurrence with summer temperatures. Once hybrids were removed, we found very little evidence of population genetic differentiation or local isolation of alleles, with high levels of gene flow, slow generation times and a lack of historical geographic isolation probably responsible for these findings.

This study has implications for our understanding of the processes involved in adaptation and assisted migration. Previous work has shown that species can not only adapt to changing environments through the development of multiple novel genetic pathways (Steane et al., 2017), but also through a process of adaptive introgression (De La Torre, Wang, Jaquish, & Aitken, 2014; Suarez‐Gonzalez, Lexer, & Cronk, 2018), whereby hybridization with congeners allows for the capture of beneficial genetic components from parent species. For example, hybrid individuals between *Picea glauca *(Moench) Voss and *Picea engelmannii *Parry ex Engelm. have been shown to utilize adaptive introgression as a way to maximize fitness in a changing climate (De La Torre, Wang et al., 2014). With environmental changes outpacing the natural ability of many species to adapt through standing genetic variation and mutation, adaptive introgression provides an alternative pathway, providing faster development of adaptive traits and rapid adaptation to novel conditions. It is in this context that we discuss our findings and explore their implications for studies of population genetics and genetically informed conservation.

### 4.1 Hybridization

Had we not considered the possibility of hybrid individuals occurring in our dataset, we would have included introgressed sites such as Wilsons Promontory and Bruny Island in the analyses, as well as 41 hybrid samples scattered through our other sites. Many of the hybrid individuals showed no obvious morphological differences (e.g., in bud and fruit shape or rough bark height and thickness) to pure specimens, with morphological approaches now generally regarded as a poor way of identifying hybrids (Field et al., 2009; McKinnon, Smith, & Potts, 2010; Rhymer & Simberloff, 1996; Schwabe, Neale, & McGlaughlin, 2015). Despite this, the Wilsons Promontory samples, which are morphologically more similar to *E. regnans* but genetically much closer to *E. obliqua*, had a different growth habit to typical *E. regnans* individuals, appearing stunted in form. This was initially thought to be the result of phenotypic plasticity rather than genetic architecture, as the trees do not resemble typical *E. obliqua* either. In our study, removing clear outliers using PCA or a similar method would have retained more than half of the hybrid samples. Including highly admixed samples and populations could have led us to make different inferences about patterns of genetic structure and possibly evolutionary history. As many modern studies of eucalypts (and other plant taxa) do not explicitly address the presence of hybridization in their study species, it is plausible that many studies are influenced by this issue. We propose that future studies always be explicit in acknowledging the potential for hybridization between their species of interest and other species, as gene flow between some species is clearly a common, and possibly evolutionarily significant, phenomenon (De La Torre, Roberts, & Aitken, 2014; Gerber, Chadoeuf, Gugerli, Lascoux, & Buiteveld, 2014; Lepais et al., 2009; Palme, Su, Palsson, & Lascoux, 2004).

Eucalypts are typically preferentially outcrossing, open‐pollinated, and often found in sympatry with multiple congeneric species, which may partly explain why more than half of all species form natural hybrid combinations (Griffin et al., 1988; Potts, Barbour, Hingston, & Vaillancourt, 2003). In addition, many of these combinations can occur at relatively high frequencies within populations (Field et al., 2009; McKinnon et al., 2010); for example, at least 27% of *Eucalyptus globulus *Labill. within 450 m of *Eucalyptus cordata *Labill. show some level of admixture (McKinnon et al., 2010). However, despite the knowledge that hybridization is not uncommon, it is often not considered in population genetic studies of eucalypts. We also note the possibility that for some species there may be multiple hybrid combinations with other species, which may vary regionally (Griffin et al., 1988). In this study, we only considered admixture with a single species; however, gene flow with red stringybark may regularly occur in particular sites within Victoria (Ashton & Sandiford, 1988).

In the case of *E. regnans*, hybridization with *E. obliqua *appears to be a more pervasive phenomenon than previously realized, with all sampled sites containing at least one hybrid individual, and two sites where more than half of the samples were hybrids. The Wilsons Promontory individuals contained very high levels of *E. obliqua* ancestry, despite their greater morphological similarity to *E. regnans*. To understand why variation in the level of admixture between populations occurs, examination of the factors controlling gene flow between eucalypt species is necessary. Previous research has identified three predominant drivers of hybridization in eucalypts, including the extent of geographic isolation, the degree of overlap in flowering times, and the level of phylogenetic divergence between species (Barbour, Potts, Vaillancourt, & Tibbits, 2006; Butcher, McDonald, & Bell, 2009; Field et al., 2009; McKinnon et al., 2010; Potts et al., 2003), although strong abiotic (e.g., climatic or geological) gradients may also be explanatory in some cases (Pryor, 1976). When stands of one species are geographically isolated from conspecifics, there is a greater opportunity for pollen from another species to successfully pollinate flowers in the isolated stand, termed “pollen swamping” (Ellstrand & Elam, 1993; Field et al., 2009). Additionally, environmental conditions can influence the degree of overlap in flowering times, with temperature previously shown to be an important driver of flowering phenology in eucalypts (Hudson, Kim, & Keatley, 2010; Law, Mackowski, Schoer, & Tweedie, 2000; Rawal, Kasel, Keatley, & Nitschke, 2015). As *E. obliqua* is a predominantly summer‐flowering species, floral development in *E. obliqua* may occur earlier in sites with warmer summers, resulting in less phenological overlap with the autumn‐flowering *E. regnans*. We therefore suggest that the primary cause of the extensive introgression of *E. obliqua* into *E. regnans *stands at Wilsons Promontory and Bruny Island is the patchy distribution of *E. regnans* in these *E. obliqua*‐dominated regions, possibly assisted by environmentally driven overlap in flowering times. The patchy distribution of *E. regnans* at these and other coastal sites (e.g., Tasman Peninsula) is likely due to their being located on the periphery of suitable climatic conditions for this species.

### 4.2 Patterns of genetic structure

The low levels of genetic population structure that we observed are not atypical for eucalypts, with a number of studies (Broadhurst, Mellick, Knerr, Li, & Supple, 2018; Dillon et al., 2015; Gauli, Steane, Vaillancourt, & Potts, 2014; Sampson et al., 2018; Supple et al., 2018) finding that geographic structure does not contribute greatly to population differentiation. Similarly, the *F*_ST_ values that we observed between sites are low but comparable to those found in other eucalypts (Sampson et al., 2018; Supple et al., 2018; Yeoh, Bell, Foley, Wallis, & Moran, 2012), and low geographic structuring of genetic diversity is not unusual in widespread forest trees, such as *Pinus taeda* L. (Eckert et al., 2010) and *Quercus lobata*Née (Grivet, Sork, Westfall, & Davis, 2008; Sork et al., 2010).

As gene flow in trees is often effected predominantly through pollen dispersal (Sork, 2016), there is often a clear difference in population structure between the nuclear genome (which is inherited biparentally) and genetic components that are inherited maternally, for example, the chloroplast in angiosperms (Sampson et al., 2018). In eucalypts, seed dispersal is highly restricted, with individual trees distributing seeds tens of metres, and stand edges typically only capable of moving about 1–2 m/year (Booth, 2017). While it is difficult to ascertain the upper limit of the dispersal curve, pollen appears to regularly disperse hundreds of metres to kilometres (Bloomfield et al., 2011; Broadhurst, 2013; Byrne, Elliott, Yates, & Coates, 2008; Sampson et al., 2018). This explains why, when investigating chloroplast structure for phylogeographic purposes, Nevill et al. (2010) found a highly structured genetic pattern in *E. regnans*, whereas our (nuclear‐based) results show very little population structuring. Chloroplast DNA is highly conserved, with genetic structure typically reflecting historical patterns of dispersal and colonization. Further, only a very small proportion of chloroplast variation is typically contained within coding regions (Young, Lanzatella, Sarath, & Tobias, 2011), suggesting that there is unlikely to be substantial levels of local selection acting on the chloroplast.

Broadhurst et al. (2017) identify range disjunctions as being one of the primary predictors for genetic differentiation within the Australian flora. This holds true for many plant and animal species across the Bass Strait, with species found in Victoria and Tasmania often being identified as genetically distinct, typically to the level of separate races or subspecies (Donsker & Gill, ; Simmons, Wapstra, & Wapstra, 2008; van Dyck, Gynther, & Baker, 2013). Even for species with low levels of isolation by distance, there are often clear Victorian and Tasmanian genetic clusters (Duncan et al., 2016). Our findings for *E. regnans*, with Gippsland and some Central Highlands sites more closely affiliated with Tasmanian sites than the Otways region, support the suggestion that, for some species, the Port Phillip Bay and surrounding area has been a bigger obstacle to gene flow than the Bass Strait since the last glacial maximum (Yeoh et al., 2012).

While the pattern of very weak genetic population structure that we observed is indicative of a lack of local isolation of alleles, local adaptation may still be occurring. With the majority of genetic variation present in the seed crop (or mature trees) of a stand, selection may be acting to promote particular genotypes within a generation, as environmental conditions filter out particular alleles. There is a steep reduction in the stem density within *E. regnans* stands in the decades after a regeneration event (von Takach Dukai, Lindenmayer, & Banks, 2018), which suggests that selection could easily promote genotypes that increase survival rates under local environmental conditions (Kuparinen, Savolainen, & Schurr, 2010). Previous studies have shown that when selection is very high, high levels of dispersal can maximize local adaptation, and when selection pressure is low (but present), local adaptation is highest under moderate levels of dispersal (Banks, Davies, & Cary, 2017; Forester, Jones, Joost, Landguth, & Lasky, 2016). This is due to migration providing the genetic diversity for selection to act upon, but also potentially overriding the effects of selective processes when selection is weak.

### 4.3 Implications for seed provenancing

Seed used for native vegetation restoration activities has historically been collected from small local geographic areas (Broadhurst et al., 2008), because of the perceived risk of introducing genotypes that are not adapted to local conditions (Hamilton, 2001). Over the past two decades, this “local is best” approach has been criticized for a number of reasons, including the failure to consider changing environmental/climatic conditions and not incorporating enough genetic variability (Broadhurst et al., 2008; Choi, 2007). By using only locally adapted genotypes, managers may be restricting the ability of populations to survive under changing conditions. To address this issue, the definition of what constitutes the most appropriate geographic spread for seed collection to encompass a beneficial amount of genetic variation needs to be considered (Breed, Stead, Ottewell, Gardner, & Lowe, 2013; Crow, Albeke, Buerkle, & Hufford, 2018; Prober et al., 2015).

As eucalypts and wattles (*Acacia* spp.) dominate the majority of Australian ecological restoration schemes (Broadhurst et al., 2015), developing knowledge for these taxa is of critical importance. Our results, and recent work by others (Bloomfield et al., 2011; Dillon et al., 2015; Supple et al., 2018), suggest that for many *Eucalyptus* species, most of the total genetic variation is held within rather than among populations. This has large implications for the concept of local provenancing. For example, Breed et al. (2013) suggest that estimates of historical gene flow such as *F*_ST_ values can be used to determine the extent of the local (*F*_ST_ < 0.05), intermediate (*F*_ST_ > 0.05 < 0.1) or distant (*F*_ST_ > 0.1 < 0.2) provenances. Under this definition, the entire range of *E. regnans *would be considered local provenance. Because most of the genetic variation of the species is already contained within local geographic areas, the source of seed used for restoration activities is not likely to be as important as previously considered. While we acknowledge that reciprocal transplant experiments show a strong effect of provenance in some species (Wang, O'Neill, & Aitken, 2010), any negative effects of using distant seed sources must be considered in light of considerable recent evidence showing that locally adapted traits can not only be maintained despite gene flow (Fitzpatrick, Gerberich, Kronenberger, Angeloni, & Funk, 2015), but also that the potential benefits of gene flow are large and often outweigh the risk of negative impacts associated with anthropogenic disturbances or novel selective pressures (Fisher, Garner, & Walker, 2009; Harrisson et al., 2016). In many cases, the preservation of genetic uniqueness and taxonomic integrity is no longer considered scientifically justifiable (Ralls et al., 2018). Further, as rare new variants are unlikely to be the cause of beneficial adaptation to local conditions (Alberto et al., 2013; Savolainen, Lascoux, & Merilä, 2013), adaptive alleles are still likely to be present in seed collected from distant localities, simply at different frequencies. Reductions in the frequency of some adaptive alleles are typically of minor importance and can be naturally corrected over a small number of generations (Fitzpatrick et al., 2015; Ralls et al., 2018). Thus, we suggest that sourcing seed from trees across a range of environments (local or distant) will ensure adaptive potential for restoration into a changing and challenging future.

There are other considerations for seed sourcing that also need to be considered. For example, climate‐adjusted provenancing has been suggested as a way of assisting plant populations with adaptation to climate change (Prober et al., 2015). To achieve this, seed sourcing for restoration would have to consider the future climate at a site and predict the best source populations to match that climate. However, there is one important consideration for this approach that has not been adequately considered in the recent literature: the issue of hybridization and introgression. The possibility that some populations contain a higher degree of admixture with another species cannot be ignored, because interspecific gene flow is one way in which species may cope with rapidly changing abiotic environments (De La Torre, Wang et al., 2014). By selecting seed from populations that have “adapted” to different climatic conditions, managers may actually be selecting seed that is simply admixed with another species. For example, our data indicate that the occurrence of *E. regnans × obliqua* hybrids is correlated with temperatures of the hottest and coolest months, so collection of seed based on temperature may inadvertently result in the collection of hybrid genotypes, or even near‐pure *E. obliqua*, which is likely not the desired outcome of management actions. It should also be noted that while our sampling avoided young trees, contemporary seed crops may show different levels of admixture in some populations. For example, those in landscapes where logging and fire have modified the extent and age structure of *E. regnans* compared to *E. obliqua*, or where changes in temperature have increased overlap in flowering times. To be certain of the hybrid status at a proposed seed collection locality, genotyping of seedlings would be necessary.

## 5 CONCLUSION

Our population genomic analysis of *E. regnans* found widespread admixture of varying levels with a congener, suggesting regular hybridization throughout the range of the species. As many genera of plants are known to form natural hybrids, it is critical that admixture and its role in the adaptive process is considered appropriately in population genetic studies, as introgressed populations may skew genetic analyses and affect management decisions. The combination of widespread hybridization and high levels of gene flow in *E. regnans, *with similar results having been found for a number of other eucalypt species, suggests that introgressive adaptation through porous genomes may be a common way for this taxon to adapt to rapid environmental change in climate and fire regimes. Selection on hybrids expressing traits harvested from sympatric congeners may allow for rapid adaptive change to new conditions. Furthermore, as the occurrence of hybrid individuals was not distributed evenly across geographic or climatic space, the use of climatic variables to select genotypes for assisted migration may not be the most appropriate way to manage eucalypts for conservation purposes and requires more detailed consideration.

## CONFLICT OF INTEREST

None declared.

## DATA ARCHIVING

Sequence data for all individuals are deposited to the NCBI Sequence Read Archive (SRA). A CSV file containing location data and called genotypes from ANGSD for all individuals are available at the Dryad Digital Repository: https://doi.org/10.5061/dryad.445m9j4.
