# Supplementary material for: Pervasive admixture between eucalypt species has consequences for conservation and assisted migration
Source: Evol Appl. 2019 Feb 12;12(4):845–60. doi: 10.1111/eva.12761 (PMC6439489; doi:10.1111/eva.12761)
Supplement: Supplementary file 1 [file EVA-12-845-s001.docx]

Supplementary material

**Table S1.** Sites visited across the natural distribution of Eucalyptus regnans in the Australian states of Victoria and Tasmania. The estimated age of trees, the number of samples collected for DNA extraction, the mean latitude and longitude and the mean elevation are also provided.

| **State** | **Region** | **Site name** | **Estimated age of trees (years)** | **No. samples** | **Latitude** | **Longitude** | **Altitude (m a.s.l.)** |
| --- | --- | --- | --- | --- | --- | --- | --- |
| Victoria | Otways | Maits Rest | 200+ | 4 | -38.75 | 143.56 | 231 |
| Victoria | Otways | Gellibrand River | 77 | 19 | -38.64 | 143.67 | 496 |
| Victoria | Otways | Erskine Falls | 77 | 21 | -38.52 | 143.92 | 339 |
| Victoria | Central Victoria | Maroondah north | 77 | 13 | -37.59 | 145.61 | 678 |
| Victoria | Central Victoria | Maroondah south | 77-200+ | 24 | -37.67 | 145.66 | 748 |
| Victoria | Central Victoria | O’Shannassy | 77-200+ | 13 | -37.67 | 145.78 | 803 |
| Victoria | Central Victoria | Powelltown | 77-90 | 25 | -37.82 | 145.85 | 724 |
| Victoria | Central Victoria | Toolangi south | 77-90 | 19 | -37.57 | 145.55 | 775 |
| Victoria | Central Victoria | Toolangi north | 50 | 20 | -37.52 | 145.50 | 701 |
| Victoria | Central Victoria | Upper Yarra north | 77 | 8 | -37.60 | 145.94 | 841 |
| Victoria | Central Victoria | Upper Yarra south | 77 | 8 | -37.77 | 146.12 | 847 |
| Victoria | Central Victoria | Dandenong Ranges | 200+ | 20 | -37.83 | 145.36 | 540 |
| Victoria | Gippsland | Wilsons Promontory | 50-60 | 21 | -39.03 | 146.40 | 268 |
| Victoria | Gippsland | Tarra Bulga | 100-250 | 21 | -38.43 | 146.57 | 607 |
| Victoria | Gippsland | Mount Elizabeth | 77 | 21 | -37.49 | 147.93 | 871 |
| Victoria | Gippsland | Errinundra Plateau | 100 | 21 | -37.28 | 148.97 | 986 |
| Tasmania | North | Dial Range | 50-70 | 21 | -41.25 | 146.07 | 211 |
| Tasmania | North | Frome | 55-65 | 20 | -41.16 | 147.90 | 291 |
| Tasmania | Central | Mount Field | 50-200+ | 21 | -42.68 | 146.71 | 450 |
| Tasmania | South | Tasman Peninsula | 55-65 | 20 | -43.10 | 147.89 | 140 |
| Tasmania | South | Arve Valley | 60-200+ | 7 | -43.13 | 146.76 | 323 |
| Tasmania | South | Bruny Island | 50+ | 20 | -43.39 | 147.31 | 242 |


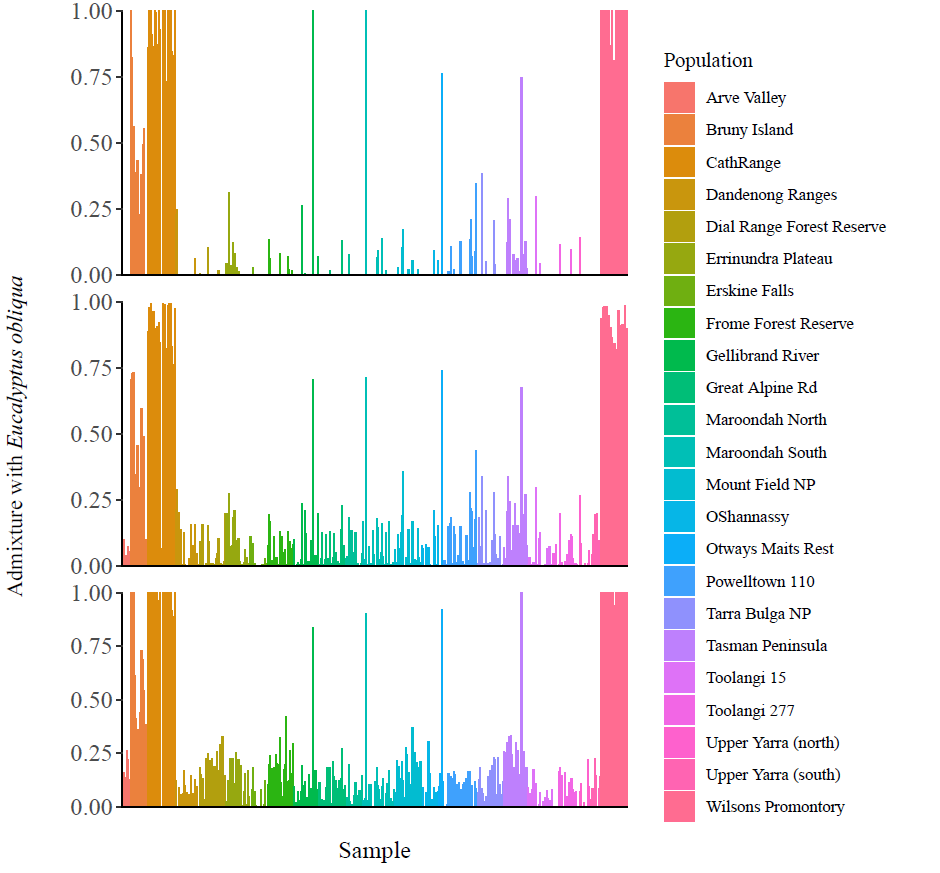


**Figure S1.** Barplots showing proportion of Eucalyptus obliqua in 21 putative E. obliqua and 359 putative Eucalyptus regnans tissue samples, genotyped using genotyping-by-sequencing. Ancestry coefficients were determined by SNPRelate (top), STRUCTURE (middle) and NGSadmix (bottom). Each individual is coloured according to the site at which it was collected. All reference E. obliqua samples belong to the CathRange population. Individuals were initially identified as either E. obliqua or E. regnans in the field using morphological characters.


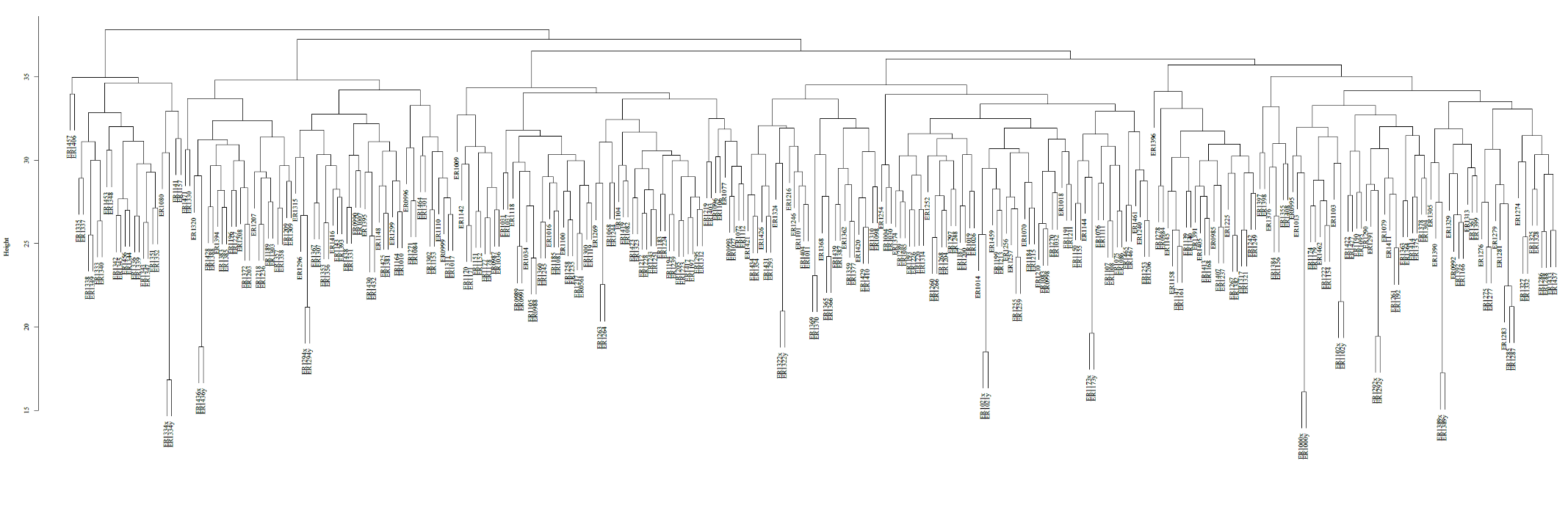


**Figure S2.** Hierarchical clustering dendrogram of 323 Eucalyptus regnans samples, including individuals admixed with Eucalyptus obliqua. Computations were done using 2474 single-nucleotide polymorphisms from across the genome. Technical replicates are identified with an x or y after the sample name and are paired together on long branches.


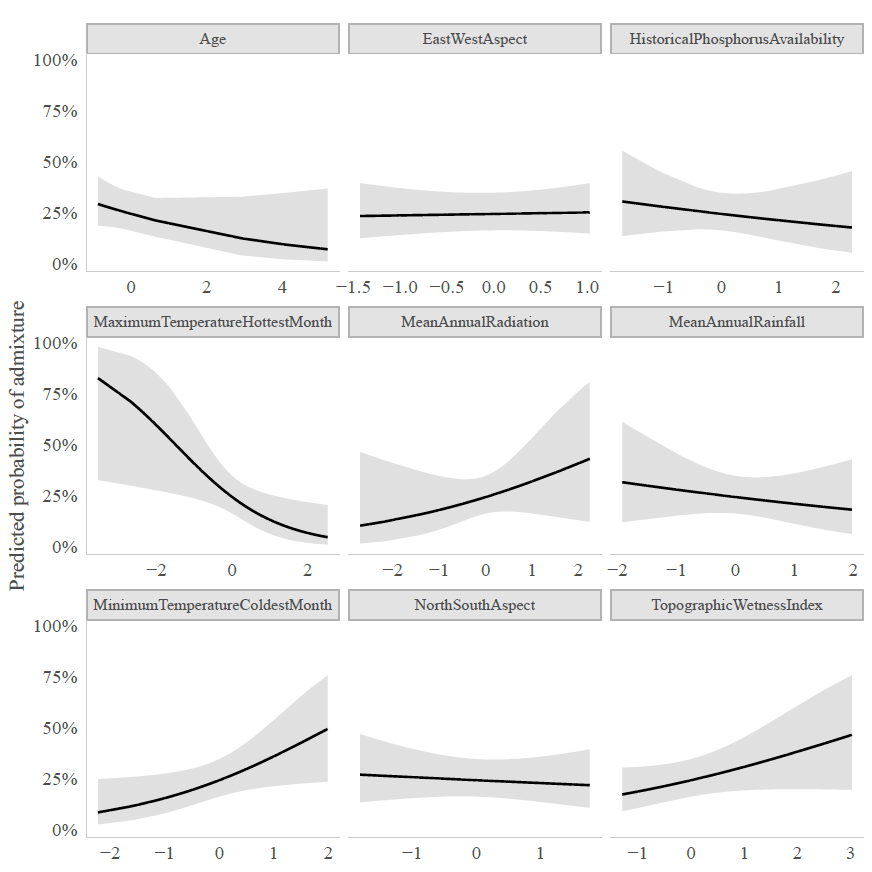


**Figure S3.** Plots showing the relationship between seven (scaled and centred) environmental variables and the predicted probability that a Eucalyptus regnans individual will be admixed (i.e. have an admixture coefficient with Eucalyptus obliqua >0.1). Results are derived from a mixed effects logistic regression. Missing data was included in the global model to rule out the possibility that it was influencing the identification of admixed individuals. Grey areas indicate 95% confidence intervals.

**Table S2.** Genetic diversity parameters (derived from 2481 single-nucleotide polymorphism markers) for 200 Eucalyptus regnans individuals in 15 populations from across the species geographic distribution. Mean values and standard errors are presented for each population, after removal of all admixed individuals.

| **Site** | **N** | ***A*** | ***A*_E_** | ***H*_E_** | ***H*_O_** | ***F*_IS_** |
| --- | --- | --- | --- | --- | --- | --- |
| Dandenong Ranges | 14 | 1.426 (0.01) | 1.122 (0.004) | 0.085 (0.003) | 0.089 (0.003) | -0.053 (0.008) |
| Dial Range | 12 | 1.363 (0.01) | 1.118 (0.004) | 0.081 (0.003) | 0.087 (0.003) | -0.08 (0.009) |
| Errinundra Plateau | 10 | 1.293 (0.011) | 1.124 (0.005) | 0.119 (0.004) | 0.096 (0.004) | -0.126 (0.01) |
| Erskine Falls | 11 | 1.341 (0.01) | 1.122 (0.004) | 0.098 (0.003) | 0.095 (0.003) | -0.111 (0.009) |
| Frome | 11 | 1.384 (0.01) | 1.127 (0.004) | 0.088 (0.003) | 0.094 (0.003) | -0.085 (0.009) |
| Gellibrand River | 13 | 1.395 (0.011) | 1.133 (0.005) | 0.113 (0.004) | 0.103 (0.004) | -0.101 (0.008) |
| Mount Elizabeth | 16 | 1.494 (0.01) | 1.13 (0.004) | 0.092 (0.003) | 0.099 (0.003) | -0.059 (0.007) |
| Maroondah north | 12 | 1.409 (0.01) | 1.123 (0.004) | 0.087 (0.003) | 0.094 (0.003) | -0.077 (0.008) |
| Maroondah south | 19 | 1.529 (0.01) | 1.121 (0.004) | 0.088 (0.002) | 0.089 (0.003) | -0.03 (0.007) |
| Mount Field | 12 | 1.397 (0.01) | 1.124 (0.004) | 0.086 (0.003) | 0.092 (0.003) | -0.073 (0.009) |
| O'Shannassy | 11 | 1.407 (0.01) | 1.123 (0.004) | 0.086 (0.003) | 0.093 (0.003) | -0.078 (0.008) |
| Powelltown | 14 | 1.463 (0.01) | 1.133 (0.004) | 0.104 (0.003) | 0.101 (0.003) | -0.066 (0.008) |
| Tarra Bulga | 14 | 1.453 (0.01) | 1.127 (0.004) | 0.09 (0.003) | 0.096 (0.003) | -0.063 (0.008) |
| Toolangi south | 15 | 1.437 (0.01) | 1.126 (0.004) | 0.1 (0.003) | 0.095 (0.003) | -0.067 (0.007) |
| Toolangi north | 16 | 1.484 (0.01) | 1.126 (0.004) | 0.094 (0.003) | 0.096 (0.003) | -0.059 (0.007) |

*N, sample size; A, number of alleles; A_E_, effective number of alleles; H_E_, expected heterozygosity; H_O_, observed heterozygosity; F*_IS_*, inbreeding coefficient.*
